# Inhibition mechanism of Ca^2+^, Mg^2+^ and Fe^3+^ in fine cassiterite flotation using octanohydroxamic acid

**DOI:** 10.1098/rsos.180158

**Published:** 2018-08-15

**Authors:** Liuyi Ren, Hang Qiu, Wenqing Qin, Ming Zhang, Yubiao Li, Penggang Wei

**Affiliations:** 1 School of Resources and Environment Engineering, Wuhan University of Technology, Wuhan 430070, People's Republic of China; 2 Hubei Key Laboratory of Mineral Resources Processing and Environment, Wuhan 430070, People's Republic of China; 3 School of Minerals Processing and Bioengineering, Central South University, Changsha 410083, People's Republic of China

**Keywords:** OHA, fine cassiterite, metal ions, flotation, inhibition

## Abstract

The existence of metal ions should not be ignored in both hydrometallurgy and flotation. In this study, the effects of Ca^2+^, Mg^2+^ and Fe^3+^ on the flotation performance of cassiterite using octanohydroxamic acid (OHA) as the collector were investigated by micro-flotation tests, X-ray photoelectron spectroscopy (XPS), Fourier transform infrared (FTIR) spectroscopy, contact angle, zeta (*ζ*) potential measurements and atomic force microscopy (AFM) imaging. The results of the flotation and contact angle experiments showed that the addition of Ca^2+^, Mg^2+^ and Fe^3+^ significantly decreased both the recovery and contact angle of cassiterite with pH ranged from 6.0 to 12.0 in the presence of OHA collector. *ζ-*Potential measurements, solution chemistry analysis and FTIR measurements indicated that the flotation recovery of the cassiterite declined due to the CaOH^+^, MgOH^+^ and Fe(OH)_3_ sites on the cassiterite surface. XPS results indicated that the chemisorption of OHA and calcium ions on the cassiterite surface finally changed its chemical properties. The AFM images also revealed that new species Fe(OH)_3_ of Fe^3+^ formed and adsorbed on the cassiterite surface at pH 9.0. The adsorption of Fe(OH)_3_ reduced the adsorption of OHA on the cassiterite surface, thus the hydrophobicity of cassiterite was deteriorated.

## 1. Introduction

Tin is a common metal used in many areas because of its high malleability, ductility and resistance to corrosion. Cassiterite is an economically important mineral of tin in the Earth's crust [1]. Gravity separation (including jigs, spirals and tables) is the dominant beneficiation strategy for the recovery of cassiterite based on the density difference between the valuable and associated gangue minerals [2–4]. However, gravity concentration is sensitive to the particle size. Once cassiterite particle size is below 40 µm, it is greatly lost in the gravity tailings [5].

Froth flotation is an effective method for separating fine target minerals from gangue minerals depending on their surface hydrophobicity differences [6,7]. Thus, flotation has been widely used in cassiterite concentration due to its advantages in fine particle processing [8–10]. Metal ions, such as Pb^2+^, Ca^2+^, Mg^2+^, Cu^2+^ and Al^3+^, commonly exist in flotation pulp due to the use of groundwater, recycled water or seawater [11]. Therefore, these metal ions influence collector adsorption and thus flotation performance [12–14]. Calcium ions are from the semi-soluble minerals such as calcite and apatite, which influence collector adsorption and flotation performance [13,15].

Previous studies indicate that metal ions adsorbed on mineral surfaces can affect flotation. Liu & Zhang [16] found that the presence of calcium ions affected the separation of chalcopyrite from galena. Liu *et al*. [17] found that the Pb^2+^ effectively improved the flotation of hemimorphite. The adsorption/precipitation of the hydrolysed species of lead cations promoted sodium oleate adsorption and formed lead oleate on the surface of hemimorphite. Potapova *et al*. [18] investigated and found that calcium ions increased the adsorption of both collector and sodium silicate on the surface of magnetite.

Alkyl or alkaryl hydroxamic acids and their salts are well-known collectors for the froth flotation of oxide minerals such as scheelite [19], rhodochrosite [20], malachite [21], pyrochlore [22], monazite [15] and cassiterite [23,24]. However, it has not been reported regarding the effect of Ca^2+^, Mg^2+^ and Fe^3+^ on cassiterite flotation when octanohydroxamic acid (OHA) is used as the collector.

In the present work, the effect of Ca^2+^, Mg^2+^ and Fe^3+^ on the flotation of pure cassiterite with OHA as the collector was intensively investigated. The experiments were carried out in a mechanical agitation flotation machine and the effects of metal ions on the adsorption of OHA on cassiterite surfaces were fundamentally investigated via *ζ*-potential determination, Fourier transform infrared (FTIR) analysis, solution chemistry analysis, X-ray photoelectron spectroscopy (XPS) analysis and atomic force microscopy (AFM) imaging.

## 2. Material and methods

### 2.1. Materials

**Figure 1.**
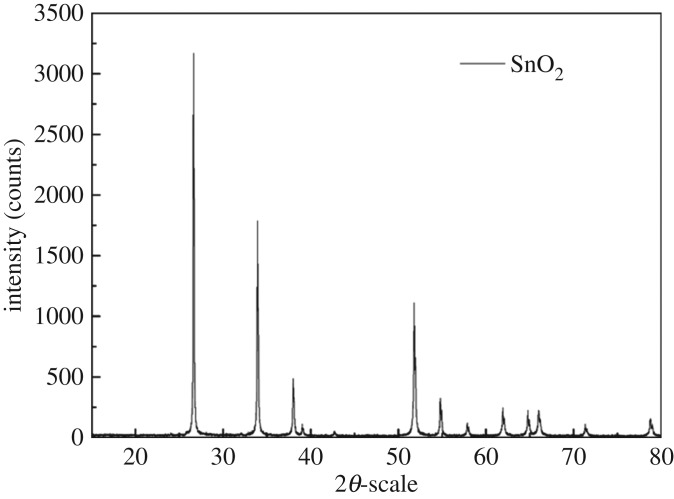
XRD patterns of the cassiterite sample.

The cassiterite samples were purchased from Yunnan Bao Yun Jewelry Company, China. The X-ray diffraction (XRD) result is shown in figure 1. The peaks of any impurities were not detected, which indicated that SnO_2_ was of high purity. Chemical analysis showed that the resultant powder contained 95.58% SnO_2_. For the micro-flotation tests, the used cassiterite samples with a particle size distribution of 20–38 µm were obtained by screening. The remaining ones were ground continuously for *ζ*-potential, XPS analysis and FTIR tests. In addition, 1 × 1 cm block samples used for the AFM experiments were finely polished and further cleaned by rinsing thoroughly with ethanol.

The OHA of 99% purity was purchased from Aladdin Bio-Chem Technology, China. CaCl_2_ was used as the source of Ca^2+^. H_2_SO_4_ and NaOH were used for pH adjustment of the suspension. All the chemicals were of analytical grade except frother pine oil (commercial grade), and distilled water was used for all experimental work.

### 2.2. Methods

#### 2.2.1. Micro-flotation

Micro-flotation tests were conducted in a mechanical agitation flotation machine with a 40 ml cell. The mixture was stirred at 1650 r.p.m. by a four-bladed impeller. In each test, 2.0 g of pure cassiterite particles were dispersed in the cell with 30 ml distilled water for 1 min. The pH was adjusted by adding a pH regulator for 3 min. The metal ions were added and the pulp was conditioned for 3 min, then the collector was introduced and conditioned for 3 min, the frother was added, followed by flotation for 4 min. Finally the flotation products were filtered, dried and weighed for recovery calculation.

#### 2.2.2. Measurements of *ζ*-potential

*ζ*-Potential measurements of cassiterite were carried out using a Zetasizer Nano ZS90 (ZEN3690, Malvern Instruments Ltd, UK). All measurements were done in a 1 mM KCl background electrolyte solution. A solid concentration of 0.05% by weight was used and conditioning procedures were similar to that used for the micro-flotation tests. The *ζ*-potential results were obtained using the average of three independent measurements with a typical variation of ±2 mV.

#### 2.2.3. X-ray photoelectron spectroscopy analysis

XPS analysis was conducted using an ESCALAB250Xi (Thermo Scientific, UK) with Al Kα X-ray source. The mineral samples were taken from flotation experiments and then washed with distilled water to a similar pH to remove any suspended colloidal particles; finally, they were introduced immediately into the fore vacuum of the spectrometers as a slurry.

#### 2.2.4. Contact angle measurements

The contact angles of cassiterite were measured using an instrument (JC2000D, Shanghai Chenzhong Digital Technology Co., Ltd). The cassiterite was treated with OHA and/or metal ions; the conditioning procedures were similar to that used for the micro-flotation tests, and modified cassiterite powder was taken from flotation experiments. The samples were vacuum-dried in a desiccator, model Sotelem, at approximately 25°C and then transferred into a tablet machine to be pressed into a wafer with a diameter of 12 mm.

#### 2.2.5. Fourier transform infrared analysis

FTIR measurements were taken on a Nicolet 6700 (Waltham, MA, USA). Pure cassiterite was ground to −5 µm for FTIR tests. Conditioning procedures were similar to that used for the flotation tests. After conditioning, the solid samples were washed three times using deionized water with the same pH and dried prior to FTIR analysis. Approximately 1% (mass fraction) of the solid sample was mixed with spectroscopic grade KBr. The analysis was conducted from 4000 to 500 cm^−1^ using 32 scans with a resolution of 4 cm^−1^.

#### 2.2.6. Atomic force microscopy imaging

Multi-mode 8 (Bruker, USA) AFM was used in the contact mode for imaging of the cassiterite surface. Triangular cantilevers with a nominal spring constant of 0.12–0.58 N m^−1^ were used for surface image measurements. The AFM measurement was commenced after the cassiterite plate had made a good contact with the chemical solution for a specific time. The images of the cassiterite surface were obtained at a scan rate of 1 Hz and a scan area of 5 × 5 µm. The images were processed offline using Nanoscope v. 5.31R1 software. Flattening and low-pass filtering were applied to remove noise.

## 3. Results and discussion

### 3.1. Micro-flotation tests

**Figure 2.**
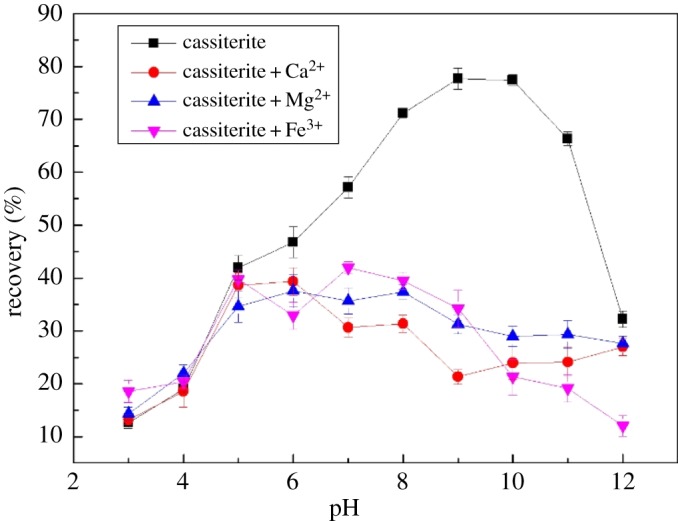
Effects of pH on cassiterite flotation when using 35 mg l^−1^ OHA and 20 mg l^−1^ metal ions.

Mineral floatability is highly related to solution pH values, which influences the component of collectors and ions in the solution. Figure 2 shows the flotation recovery of cassiterite versus pH at a collector dosage of 35 mg l^−1^ and metal ion concentration of 20 mg l^−1^. The maximum recovery, 77.63%, was obtained at a pH of 9.0 in the absence of metal ions. The floatability of cassiterite was significantly depressed by Ca^2+^, Mg^2+^ and Fe^3+^, while the pH ranged from 6 to 12.

**Figure 3.**
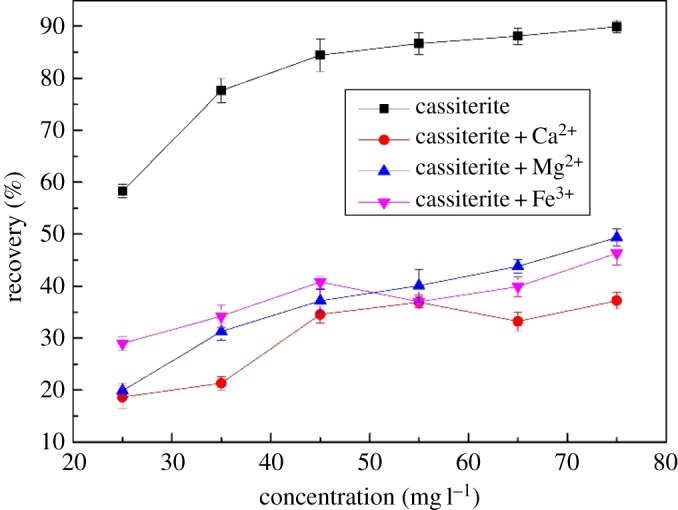
Effects of OHA concentration on cassiterite flotation at a pH of 9.0.

With the solution pH maintained at a value of 9.0, figure 3 shows the flotation recovery of cassiterite with a collector concentration from 25 to 75 mg l^−1^ and metal ion concentration of 20 mg l^−1^. The recovery of cassiterite approached 90% at high collector dosage levels without metal ions. As can be seen, the floatability of cassiterite decreased significantly in the presence of the metal ions.

**Figure 4.**
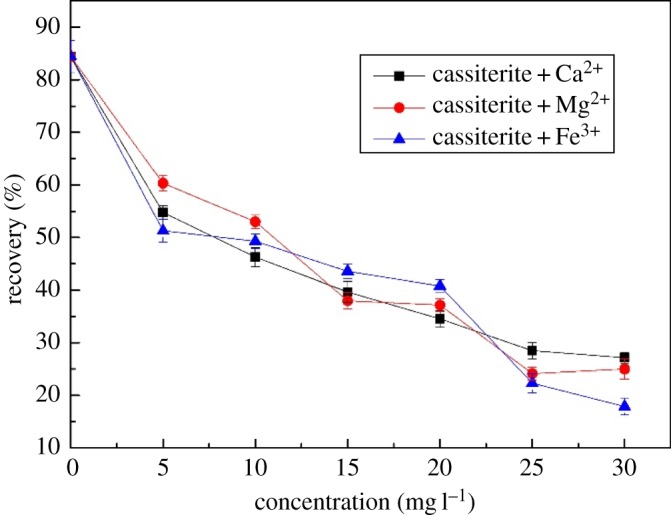
Flotation recovery as a function of metal ions concentration at a pH of 9.0 with 45 mg l^−1^ OHA.

Figure 4 shows the effect of metal ion concentration on the flotation of cassiterite in the presence of 45 mg l^−1^ OHA dosage at a pH of 9.0. Cassiterite recovery decreased exponentially with an increase in metal ions. The recovery of cassiterite dropped from 84.42% to 27.14%, 24.98% and 17.88% with an elevation in the Ca^2+^, Mg^2+^ and Fe^3+^ concentrations, respectively. Therefore, the Ca^2+^, Mg^2+^ and Fe^3+^ cations present in the pulp significantly reduced the floatability of cassiterite, even at a pH range of 6–12.

### 3.2. Contact angle measurement

**Figure 5.**
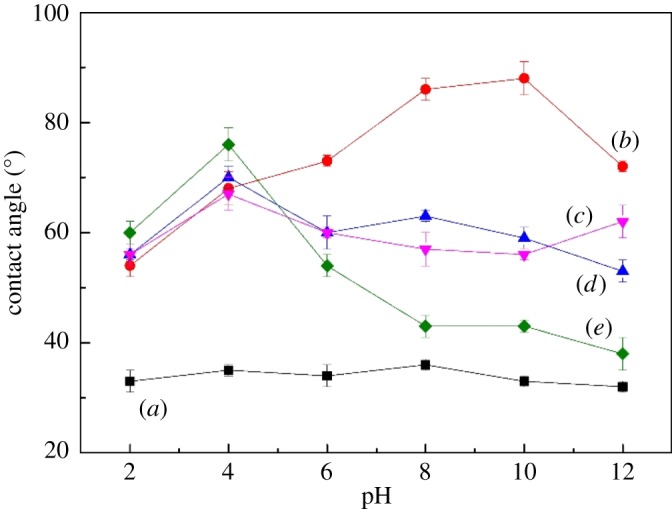
Contact angles of cassiterite as a function of pH: (*a*) cassiterite alone, (*b*) cassiterite in the presence of 45 mg l^−1^ OHA, (*c*) cassiterite in the presence of 45 mg l^−1^ OHA and 20 mg l^−1^ Ca^2+^, (*d*) cassiterite in the presence of 45 mg l^−1^ OHA and 20 mg l^−1^ Mg^2+^, and (*e*) cassiterite in the presence of 45 mg l^−1^ OHA and 20 mg l^−1^ Fe^3+^.

The contact angle is useful for the determination of the wetting characteristic and floatability of a solid mineral [25]. The contact angles of cassiterite are depicted in figure 5. Before the interaction between cassiterite and OHA, the surface of the cassiterite was completely hydrophilic, and values of the contact angle were approximately 35°. The contact angle of the cassiterite sample increased to a range of 54–88° after its interaction with OHA. Moreover, the contact angle decreased in the presence of OHA and metal ions. For pH ranged from 6.0 to 12.0, the contact angles became lower, demonstrating a stronger interaction between the cassiterite cells and OHA/metal ions in this pH range.

The results in figure 5 illustrate that the cassiterite was completely hydrophilic in the absence of both OHA and metal ions, while the addition of OHA enhanced the contact angle. The addition of metal ions made the contact angle significantly decrease with pH ranged from 6.0 to 12.0. This indicated that the hydrophobicity of cassiterite increased by the addition of OHA, and then decreased if both metal ions and OHA were added. The decrease in the contact angle and hydrophobicity of the cassiterite surface after the addition of metal ions were caused by the reduced adsorption of the OHA. This agrees with the results of the flotation recovery of cassiterite as shown in figure 3.

### 3.3. *ζ*-Potential measurements and solution chemistry analysis

**Figure 6.**
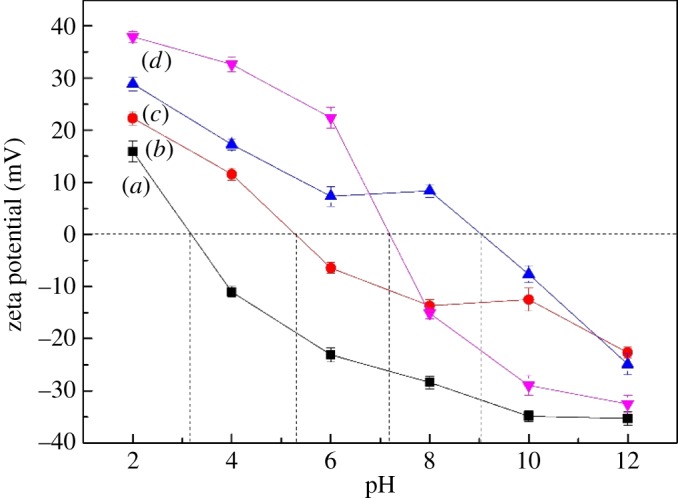
*ζ*-Potential of cassiterite as a function of pH: (*a*) cassiterite alone; (*b*) cassiterite in the presence of 20 mg l^−1^ Ca^2+^; (*c*) cassiterite in the presence of 20 mg l^−1^ Mg^2+^; (*d*) cassiterite in the presence of 20 mg l^−1^ Fe^3+^.

Electrokinetic phenomena play an important role in understanding the flotation phenomena based on the isoelectric point (IEP) of mineral particles. The surface charge and surface potential determine the particle–chemical interactions (e.g. collector adsorption) and particle–particle interactions (e.g. hetero-aggregation) [26]. Figure 6 shows the *ζ*-potential of cassiterite as a function of pH in the presence of metal ions, and the IEP of cassiterite alone was located at a solution pH of 3.2, which is lower compared with that of 4.2, 5.0 reported in the literature [10,24,27]. It may be caused by the absorption of SO_4_^2−^ on the surface of cassiterite and hence the IEP of cassiterite is influenced [28]. In the presence of Ca^2+^, Mg^2+^ and Fe^3+^, the *ζ*-potential of the cassiterite was increased significantly and the IEP of cassiterite increased from a pH of 3.2 to 5.3, 9.1 and 7.2, respectively. The influence of Ca^2+^ and Mg^2+^ on the electrokinetic properties of cassiterite was more evident in basic solutions, while that of Fe^3+^ was more evident in acidic solutions. The significant increase in the IEP of cassiterite provided evidence of metal ions adsorbing/precipitating onto the negatively charged cassiterite surfaces.

**Figure 7.**
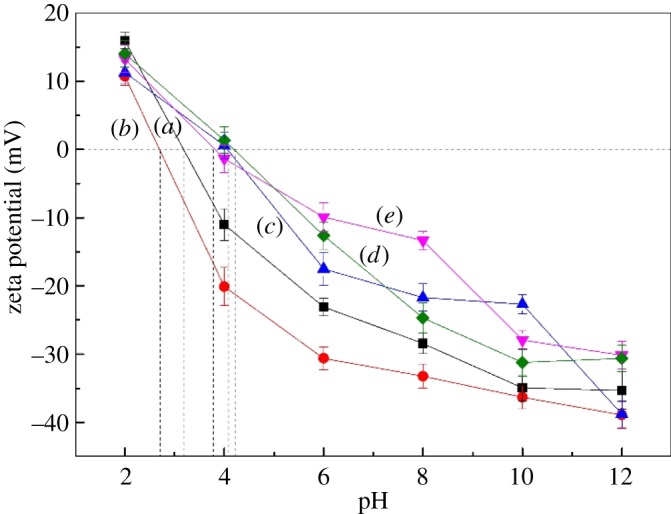
*ζ*-Potential of cassiterite as a function of pH: (*a*) cassiterite alone; (*b*) cassiterite in the presence of 45 mg l^−1^ OHA; (*c*) cassiterite in the presence of 45 mg l^−1^ OHA and 20 mg l^−1^ Ca^2+^; (*d*) cassiterite in the presence of 45 mg l^−1^ OHA and 20 mg l^−1^ Mg^2+^; (*e*) cassiterite in the presence of 45 mg l^−1^ OHA and 20 mg l^−1^ Fe^3+^.

Figure 7 shows the *ζ*-potentials of cassiterite as a function of pH in the presence or absence of both metal ions and OHA. The IEP of cassiterite decreased from a pH of 3.2 to 2.7 after OHA was added into the pulp suspension. We can attribute the decrease in the IEP values to a specific adsorption of anionic collector OHA on the cassiterite surface. Moreover, in the presence of OHA and Ca^2+^, Mg^2+^ and Fe^3+^, the IEP of the cassiterite was still increased significantly, which varied from a pH of 2.7 to 4.1, 3.8 and 4.2, respectively, even though the adsorption of OHA had a negative effect on the IEP. This indicated that the presence of metal ions in the solution played a more important role in determining the IEP of the cassiterite than the OHA.

**Figure 8.**
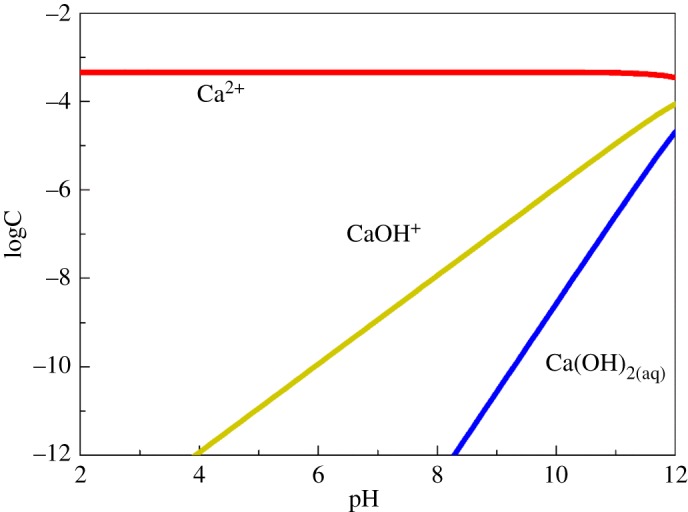
Specification diagram of calcium ions in solution.

The equilibrium concentrations of Ca^2+^, CaOH^+^ and Ca(OH)_2(aq)_ in a solution containing 20 mg l^−1^ (1.8 × 10^−4^ mol l^−1^) of total calcium ions were calculated and are shown in figure 8. Ca^2+^ was the dominant species at the pH range of 3–12, while concentrations of CaOH^+^ increased with a rise of the solution pH value. At the pH range of 8–10, the *ζ*-potential of cassiterite in the presence of Ca^2+^ increased significantly compared to the cassiterite alone, which can be attributed to the adsorption of positively charged CaOH^+^ on the cassiterite surface. In addition, micro-flotation tests in figure 3 indicate that cassiterite flotation was inhibited at a pH of 9.0 in the presence of 20 mg l^−1^ Ca^2+^. It is reasonable to suggest that the cassiterite surface was covered with a layer of CaOH^+^ attached via hydrogen bonding and electrostatic attraction [15].

**Figure 9.**
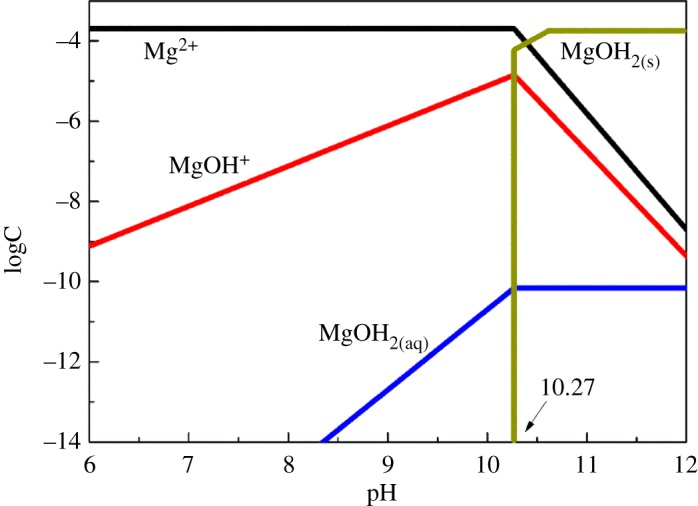
Specification diagram of magnesium ions in solution.

The equilibrium concentrations of Mg^2+^, MgOH^+^ and Mg(OH)_2(aq)_ in a solution containing 20 mg l^−1^ (2.1 × 10^−4^ mol l^−1^) of total magnesium ions were calculated and are shown in figure 9. As can be seen, Mg^2+^ existed as free ions throughout the pH range of 2.0–10.0; concentrations of MgOH^+^ increased significantly with the increase in pH; magnesium ions were predominantly in the form of Mg(OH)_2(s)_ when pH > 10.27. The *ζ*-potential results showed that the influence of Mg^2+^ on the electrokinetic properties of cassiterite was more evident in basic solutions. Combined with flotation results, it was determined that hydroxy complexes (MgOH^+^) and precipitates (Mg(OH)_2_) could adsorb onto the mineral surface.

**Figure 10.**
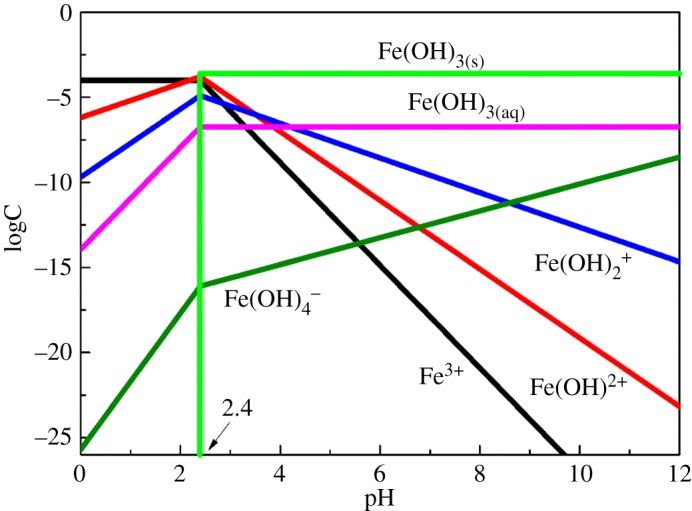
Specification diagram of iron ions in solution.

Similarly, the species distribution of 20 mg l^−1^ (1.2 × 10^−4^ mol l^−1^) Fe^3+^ was calculated, and the result is shown in figure 10. As can be seen, the positively charged Fe^3+^, Fe(OH)^2+^ and Fe( OH)_2_^+^ species all existed across the acidic pH range. The *ζ*-potential tests in figure 6 show that the addition of Fe^3+^ changed the surface charges of cassiterite from negative to positive at an acidic pH range, which indicated that the species of Fe^3+^, Fe(OH)^2+^ and Fe(OH)_2_^+^ were expected to adsorb on the cassiterite mineral surfaces at this pH range. Iron ions were predominantly in the form of Fe(OH)_2(s)_ and Fe(OH)_4_^−^ when pH > 8. According to the theory of electrostatic attraction, a positive surface can attract a negatively charged species. The flotation of cassiterite should also be enhanced in the presence of Fe^3+^ [29]. However, micro-flotation tests here indicated that the cassiterite flotation using OHA as the collector was inhibited in the presence of Fe^3+^. It was determined that Fe(OH)_3_ colloids adsorbed on the cassiterite surfaces, while Fe(OH)_3(s)_ had similar zeta potentials to that of cassiterite measured with Fe^3+^ alone [30].

### 3.4. Fourier transform infrared analysis

**Figure 11.**
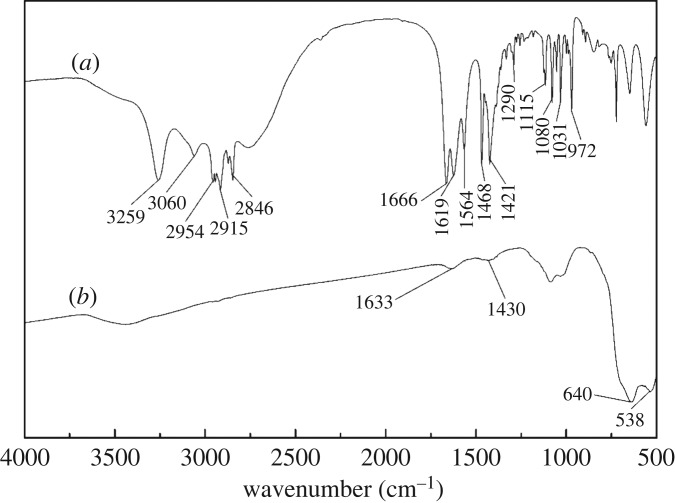
FTIR spectra of cassiterite and OHA: (*a*) OHA; (*b*) cassiterite conditioned in water at a pH of 9.0.

FTIR analysis was used to evaluate the chemical changes of cassiterite surfaces before and after the adsorption of OHA. Figure 11*a* shows the spectrum of OHA. The peak at 3259 cm^−1^ was the N–N stretch, and the peak at 3060 cm^−1^ was the Fermi resonance of the C–N stretch and the N–H bend. The peaks at 2954 and 2915 cm^−1^ were the C–H antisymmetric stretch for CH_2_ and CH_3_, and the peak at 2846 cm^−1^ was due to the C–H symmetric stretch for CH_2_/CH_3_. The peaks at 1666 and 1619 cm^−1^ were the C=O stretches. The bands at 1564 and 1468 cm^−1^ were related to the C–N stretch. The bands at 1421 and 1115 cm^−1^ were related to the N–O–H bend and C–H symmetric deformation, respectively. The peak at 1080 cm^−1^ resulted from the C–C–C stretch. The bands at 1031 and 972 cm^−1^ were attributed to the C–O stretch and the N–O stretch, respectively. Figure 11*b* shows the spectrum of cassiterite. The bands of cassiterite at 640 and 538 cm^−1^ are assigned to the antisymmetric vibration peak of Sn–O.

**Figure 12.**
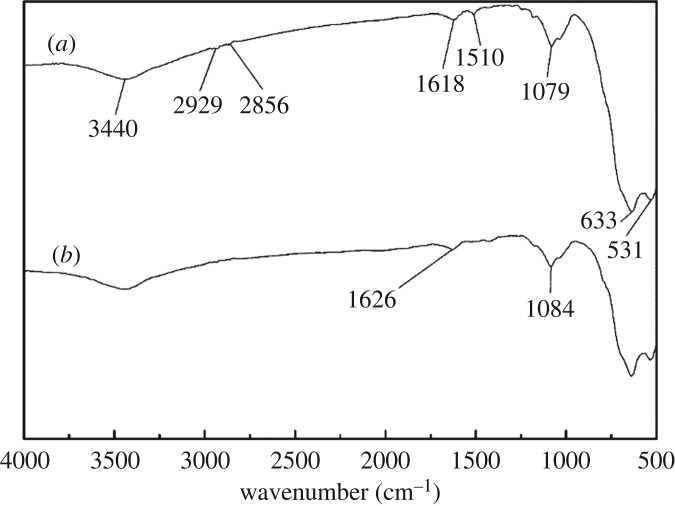
Effect of Ca^2+^ on the adsorption of OHA on cassiterite: (*a*) cassiterite conditioned in OHA at a pH of 9.0; (*b*) cassiterite conditioned in Ca^2+^ and OHA.

**Figure 13.**
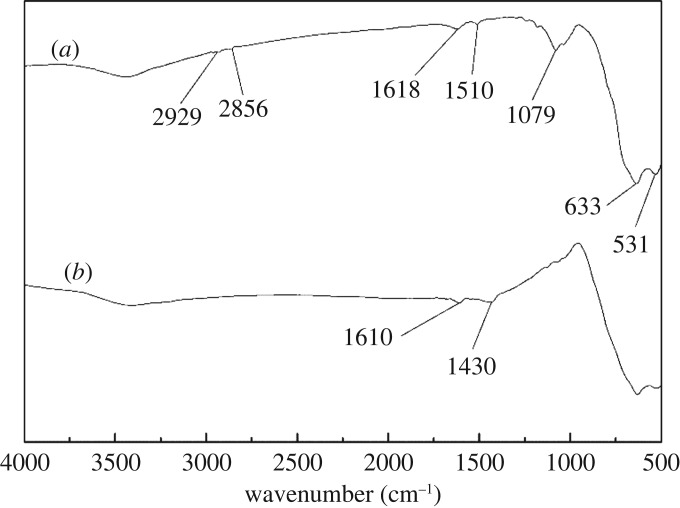
Effect of Mg^2+^ on the adsorption of OHA on cassiterite: (*a*) cassiterite conditioned in OHA at a pH of 9.0; (*b*) cassiterite conditioned in Mg^2+^ and OHA.

**Figure 14.**
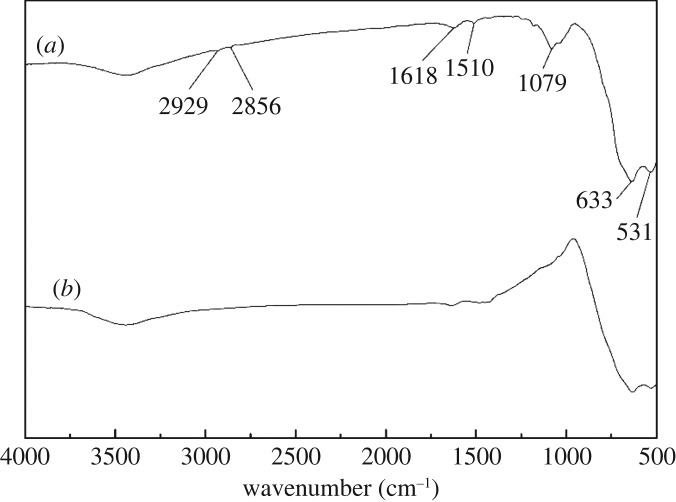
Effect of Fe^3+^ on the adsorption of OHA on cassiterite: (*a*) cassiterite conditioned in OHA at a pH of 9.0; (*b*) cassiterite conditioned in Fe^3+^ and OHA.

FTIR spectra of cassiterite with OHA at a pH of 9.0 in the presence and absence of Ca^2+^, Mg^2+^ and Fe^3+^ are plotted in figures 12–14, respectively. As can be seen from figure 12*a*, after the adsorption of OHA, new bands at 1510 and 1618 cm^−1^ appeared, indicating the occurrence of chemical adsorption. OHA spectra showed that the peaks at 1666, 1619 and 1564 cm^−1^ represented the C=O stretch and the C–N stretch. It is reasonable to suggest that the chemical reaction which occurred between OHA and cassiterite involved the C–N and C=O groups. As can be seen from figure 12*b*, when Ca^2+^ was added to the cassiterite system, the peak at 1618 cm^−1^ shifted to 1626 cm^−1^ and the peak at 1510 cm^−1^ disappeared. Figure 13*b* shows that the peak at 1618 cm^−1^ shifted to 1610 cm^−1^ and the peak at 1510 cm^−1^ shifted to 1430 cm^−1^ in the presence of Mg^2+^. Figure 14*b* shows that both the peaks at 1618 and 1510 cm^−1^ disappeared in the presence of Fe^3+^. It could be inferred that the presence of additional species on the surface of cassiterite can be detected upon the addition of Ca^2+^, Mg^2+^ and Fe^3+^.

### 3.5. X-ray photoelectron spectroscopy analysis

**Figure 15.**
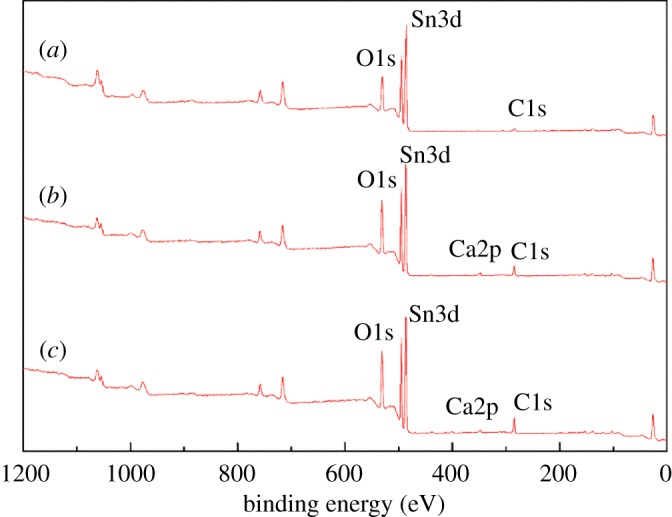
XPS survey spectra of (*a*) untreated cassiterite, (*b*) cassiterite treated with calcium ions alone, and (*c*) cassiterite treated with both calcium ions and OHA.

XPS is one of the most useful techniques for surface analysis in order to obtain chemical information [31]. XPS analysis was also employed in this investigation, to further explore the surface properties of cassiterite. The XPS survey spectra of cassiterite, cassiterite treated with calcium ions and cassiterite treated with both calcium ions and OHA are shown in figure 15. In figure 15*a*, no Ca signal is displayed in the XPS survey spectra of untreated cassiterite, indicating the high purity of the cassiterite samples. For the cassiterite treated with calcium ions, the Ca spectral peak is displayed in figure 15*b*,*c*, which indicates that the calcium ions interacted with the mineral surface.

**Figure 16.**
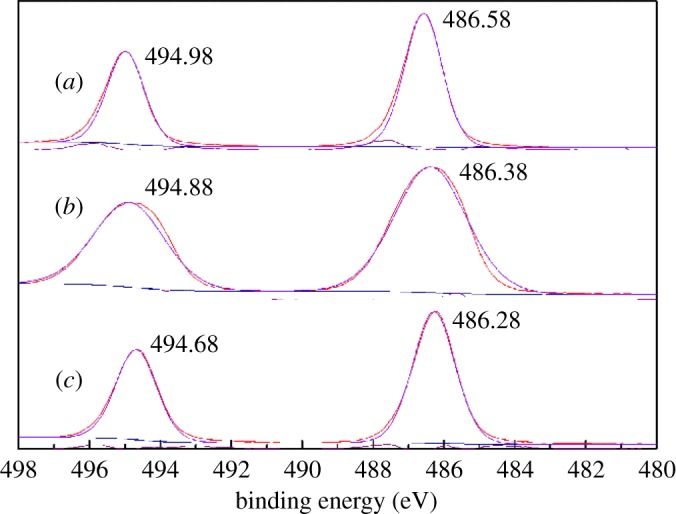
Sn3d XPS spectra of (*a*) cassiterite alone; (*b*) cassiterite in the presence of 20 mg l^−1^ Ca^2+^; (*c*) cassiterite in the presence of 45 mg l^−1^ OHA and 20 mg l^−1^ Ca^2+^.

Figure 16 shows the binding energies of the Sn3d electrons of cassiterite alone, cassiterite treated with calcium ions and cassiterite treated with calcium ions and OHA. In figure 16*a*, there are two pairs of Sn3d doublets, with a binding energy of 486.58 eV for Sn3d_5/2_ and 494.98 eV for Sn3d_3/2_. These peaks are ascribed to the Sn species from cassiterite surfaces [32]. As shown in figure 16*b*, the binding energies of Sn3d_5/2_ and Sn3d_3/2_ on a calcium ion-treated cassiterite surface were decreased by 0.1 eV and 0.2 eV, respectively. Moreover, the binding energies of Sn3d_5/2_ and Sn3d_3/2_ on the cassiterite surfaces treated by calcium ions and OHA (figure 16*c*) were decreased by 0.2 eV and 0.1 eV, respectively, compared to the one treated with calcium ions. These results indicate that a weak interaction occurred between the calcium ions and Sn sites on the cassiterite surface, and the Sn sites on the cassiterite surfaces exhibited low reactivity [29,33].

**Figure 17.**
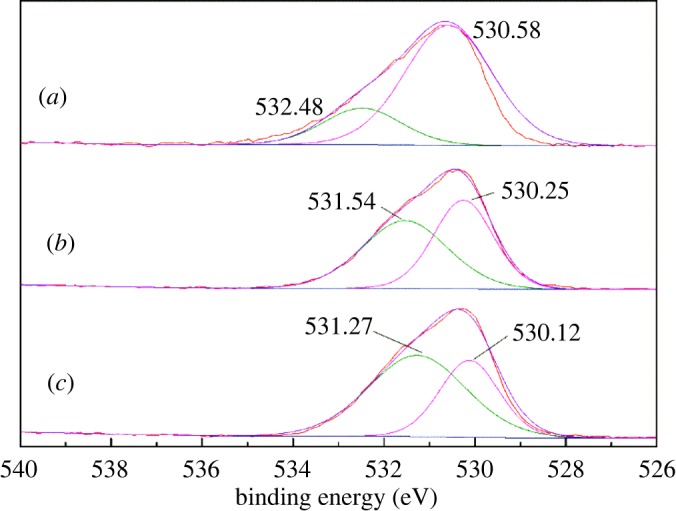
O1s XPS spectra of (*a*) cassiterite alone; (*b*) cassiterite in the presence of 20 mg l^−1^ Ca^2+^; (*c*) cassiterite in the presence of 45 mg l^−1^ OHA and 20 mg l^−1^ Ca^2+^.

Figure 17 shows the binding energies of the O1s electrons of cassiterite alone, cassiterite treated with calcium ions and cassiterite treated with calcium ions and OHA. There are two spectral peaks at 530.58 and 532.48 eV on the spectrum of cassiterite alone; the peak at 530.58 eV was assigned to the oxygen in the cassiterite and the peak at 532.48 eV was attributed to the oxygen in the hydroxyl species [33,34]. The binding energy of the O1s spectrum on the calcium ion-treated cassiterite surface was decreased by 0.33 eV, which indicates that the electronic environment of O atoms was changed. As shown in figure 17*c*, the O1s spectrum also displayed two peaks in the presence of OHA and calcium ions. The peak at 531.27 eV was assigned to the O=C oxygen atom of the OHA molecule [35,36]; it showed the adsorption of OHA on the surface of cassiterite. The peak at 530.12 eV was assigned to the oxygen of the cassiterite; the binding energy of this peak decreased by 0.13 eV compared to the calcium ion-treated cassiterite surface and 0.46 eV compared to the untreated cassiterite surface. This phenomenon indicated that the chemical environment of O1s changed under the combined actions of calcium ions and OHA on the cassiterite surface.

### 3.6. Atomic force microscopy imaging

**Figure 18.**
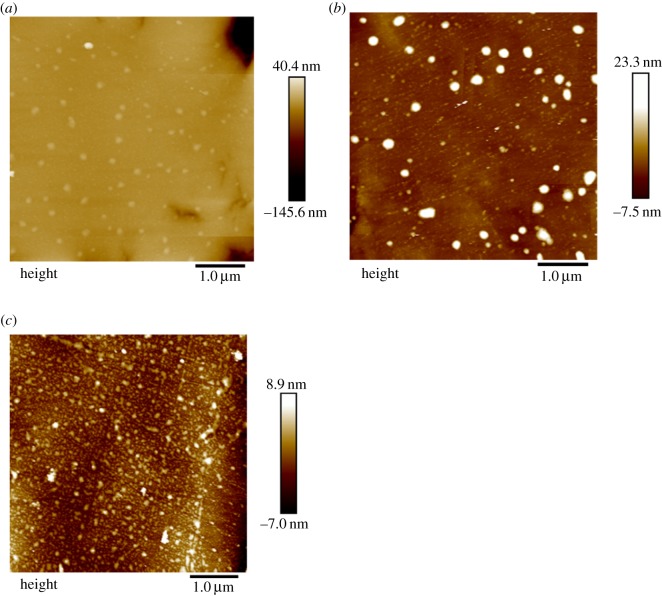
AFM images of (*a*) freshly cleaved cassiterite, (*b*) cassiterite in the presence of 45 mg l^−1^ OHA at a pH of 9.0, (*c*) cassiterite in the presence of 45 mg l^−1^ OHA and 20 mg l^−1^ Fe^3+^ at pH of 9.0.

AFM is a powerful tool for surface chemistry research. It can describe the surface topography without damaging the delicate surface. In this study, AFM was used to investigate the adsorption of OHA and Fe^3+^ on cassiterite surfaces. The AFM images of OHA and Fe^3+^ adsorbed on cassiterite are shown in figure 18.

As revealed in figure 18*a*, the fresh cassiterite surface was flat and smooth except one small bright area and another dark area. In figure 18*b*, many small patches appeared on the cassiterite surface after the conditioning with 45 mg l^−1^ OHA. It was certified that OHA directly adsorbed on the mineral surface. This agreed well with the FTIR analysis (figure 11*a*). When Fe^3+^ was added during the conditioning, it was expected that Fe^3+^ would adsorb on the cassiterite surface. As shown in figure 18*c*, the surface became rougher than that of pure cassiterite and the cassiterite surface after OHA conditioning. However, the height of figure 18*c* is lower than that of figure 18*b*. The reason for this may be the adsorption of Fe^3+^ on the cassiterite surface reducing the adsorption of OHA on the cassiterite surface. The results of the chemical calculation of solution in figure 9 showed that Fe(OH)_3(s)_ was the dominant species at a pH of 9.0. It was concluded that the Fe(OH)_3_ colloids impeded the adsorption of the collector and made the cassiterite surface hydrophilic. It also indicated that the surface of cassiterite was covered with Fe(OH)_3(s)_, and then a hydrophilic film formed which prevented the adsorption of OHA. Thus, the flotation was inhibited.

## 4. Conclusion

The effect of Ca^2+^, Mg^2+^ and Fe^3+^ on flotation of cassiterite was investigated in this study. Flotation results showed that the recovery of cassiterite dropped from 84.42% to 27.14%, 24.98% and 17.88% in the presence of 30 mg l^−1^ Ca^2+^, Mg^2+^ and Fe^3+^, respectively. The presence of Ca^2+^, Mg^2+^ and Fe^3+^ cations in the pulp significantly reduced the floatability of cassiterite at pH ranging from 6 to 12. Contact angle results indicated that the contact angle of cassiterite decreased in the presence of both OHA and metal ions compared to OHA alone at the pH range of 6–12. The decrease of the contact angle was the basis of the decrease in cassiterite recovery in flotation experiments. *ζ*-potential, FTIR measurements and solution chemistry analysis revealed that the addition of Ca^2+^, Mg^2+^ and Fe^3+^ in the flotation pulp produced some additional species on the surface of cassiterite. The new sites including CaOH^+^, MgOH^+^ and Fe(OH)_3_ on the cassiterite surface decreased the recovery of cassiterite. In addition, XPS results indicated that tin and oxygen atoms on the cassiterite mineral surface were changed under the combined action of calcium ions and OHA. AFM images offered direct evidence that the Fe(OH)_3_ colloids formed on the surface of cassiterite at a pH of 9.0 impeded the adsorption of OHA and made the cassiterite surface hydrophilic.

## Supplementary Material

original data and descriptions

## Data accessibility

Our data and descriptions were uploaded as electronic supplementary material.

## References

[1] Sun L, Hu Y, Sun W, Gao Z, Tian M 2017 Selective recovery of mushistonite from gravity tailings of copper–tin minerals in Tajikistan. Minerals 7, 242 (10.3390/min7120242)

[2] Angadi SI, Sreenivas T, Jeon HS, Baek SH, Mishra BK 2015 A review of cassiterite beneficiation fundamentals and plant practices. Miner. Eng. 70, 178–200. (10.1016/j.mineng.2014.09.009)

[3] Angadi SI, Eswaraiah C, Jeon HS, Mishra BK, Miller JD 2017 Selection of gravity separators for the beneficiation of the Uljin tin ore. Miner. Process. Extr. Metall. Rev. 38, 54–61. (10.1080/08827508.2016.1262856)

[4] Leistner T, Embrechts M, Leißner T, Chelgani SC, Osbahr I, Möckel R, Peuker UA, Rudolph M 2016 A study of the reprocessing of fine and ultrafine cassiterite from gravity tailing residues by using various flotation techniques. Miner. Eng. 96–97, 94–98. (10.1016/j.mineng.2016.06.020)

[5] De Ruijter MA 1979 Particle size effects in the flotation of cassiterite. Master's thesis, University of the Witwatersrand, Johannesburg, South Africa 12–13.

[6] Liu J, Wang Y, Luo D, Zeng Y 2018 Use of ZnSO**_4_** and SDD mixture as sphalerite depressant in copper flotation. Miner. Eng. 121, 31–38. (10.1016/j.mineng.2018.03.003)

[7] Liu J, Wang Y, Luo D, Chen L, Deng J 2018 Comparative study on the copper activation and xanthate adsorption on sphalerite and marmatite surfaces. Appl. Surf. Sci. 439, 263–271. (10.1016/j.apsusc.2018.01.032)

[8] Chelgani SC, Leistner T, Rudolph M 2015 Investigating the recovery of ultrafine cassiterite from tailings disposals using oil-assisted flotation methods. In *7th Int. Flotation Conf. (Flotation ’15), Cape Town, South Africa, 16–19 November*. Red Hook, NY: Curran Associates.

[9] Zhou Y, Tong X, Song S, Wang X, Deng Z, Xie X 2014 Beneficiation of cassiterite fines from a tin tailing slime by froth flotation. Sep. Sci. Technol. 49, 458–463. (10.1080/01496395.2013.818036)

[10] Sreenivas T, Padmanabhan NPH 2002 Surface chemistry and flotation of cassiterite with alkyl hydroxamates. Colloids Surf. A Physicochem. Eng. Asp. 205, 47–59. (10.1016/S0927-7757(01)01146-3)

[11] Hirajima T, Suyantara GPW, Ichikawa O, Elmahdy AM, Miki H, Sasaki K 2016 Effect of Mg**^2+^** and Ca**^2+^** as divalent seawater cations on the floatability of molybdenite and chalcopyrite. Miner. Eng. 96–97, 83–93. (10.1016/j.mineng.2016.06.023)

[12] Deng J, Wen S, Xian Y, Liu J, Bai S 2013 New discovery of unavoidable ions source in chalcopyrite flotation pulp: fluid inclusions. Miner. Eng. 42, 22–28. (10.1016/j.mineng.2012.10.010)

[13] Ejtemaei M, Irannajad M, Gharabaghi M 2012 Role of dissolved mineral species in selective flotation of smithsonite from quartz using oleate as collector. Int. J. Miner. Process. 114–117, 40–47. (10.1016/j.minpro.2012.09.004)

[14] Horta D, Monte MBDM, Filho ELDSL 2016 The effect of dissolution kinetics on flotation response of apatite with sodium oleate. Int. J. Miner. Process. 146, 97–104. (10.1016/j.minpro.2015.12.003)

[15] Zhang W, Honaker RQ, Groppo JG 2017 Flotation of monazite in the presence of calcite part I: calcium ion effects on the adsorption of hydroxamic acid. Miner. Eng. 100, 40–48. (10.1016/j.mineng.2016.09.020)

[16] Liu Q, Zhang Y 2000 Effect of calcium ions and citric acid on the flotation separation of chalcopyrite from galena using dextrin. Miner. Eng. 13, 1405–1416. (10.1016/S0892-6875(00)00122-9)

[17] Liu C, Feng Q, Zhang G, Ma W, Meng Q, Chen Y 2016 Effects of lead ions on the flotation of hemimorphite using sodium oleate. Miner. Eng. 89, 163–167. (10.1016/j.mineng.2016.02.002)

[18] Potapova E, Grahn M, Holmgren A, Hedlund J 2010 The effect of calcium ions and sodium silicate on the adsorption of a model anionic flotation collector on magnetite studied by ATR-FTIR spectroscopy. J. Colloid Interface Sci. 345, 96–102. (10.1016/j.jcis.2010.01.056) 20153478

[19] Zhao G, Zhong H, Qiu X, Wang S, Gao Y, Dai Z, Huang J, Liu G 2013 The DFT study of cyclohexyl hydroxamic acid as a collector in scheelite flotation. Miner. Eng. 49, 54–60. (10.1016/j.mineng.2013.04.025)

[20] Zhou F, Yan C, Wang H, Sun Q, Wang Q, Alshameri A 2015 Flotation behavior of four C18 hydroxamic acids as collectors of rhodochrosite. Miner. Eng. 78, 15–20. (10.1016/j.mineng.2015.04.006)

[21] Marion C, Jordens A, Li R, Rudolph M, Waters KE 2017 An evaluation of hydroxamate collectors for malachite flotation. Sep. Purif. Technol. 183, 258–269. (10.1016/j.seppur.2017.02.056)

[22] Gibson CE, Kelebek S, Aghamirian M, Yu B 2015 Flotation of pyrochlore from low grade carbonatite gravity tailings with benzohydroxamic acid. Miner. Eng. 71, 97–104. (10.1016/j.mineng.2014.11.006)

[23] Wu XQ, Zhu JG 2006 Selective flotation of cassiterite with benzohydroxamic acid. Miner. Eng. 19, 1410–1417. (10.1016/j.mineng.2006.02.003)

[24] Sreenivas T, Manohar C 2000 Adsorption of octyl hydroxamic acid/salt on cassiterite. Miner. Process. Extr. Metall. Rev. 20, 503–519. (10.1080/08827500008547441)

[25] Chau TT 2009 A review of techniques for measurement of contact angles and their applicability on mineral surfaces. Miner. Eng. 22, 213–219. (10.1016/j.mineng.2008.07.009)

[26] Alvarez-Silva M, Uribe-Salas A, Waters KE, Finch JA 2016 Zeta potential study of pentlandite in the presence of serpentine and dissolved mineral species. Miner. Eng. 85, 66–71. (10.1016/j.mineng.2015.10.018)

[27] Xu Y, Qin W 2012 Surface analysis of cassiterite with sodium oleate in aqueous solution. Sep. Sci. Technol. 47, 502–506. (10.1080/01496395.2011.617352)

[28] Qin W, Ren L, Xu Y, Wang P, Ma X 2012 Adsorption mechanism of mixed salicylhydroxamicacid and tributylphosphate collectors in fine cassiterite electro-flotation system. J. Central South Univ. 19, 1711–1717. (10.1007/s11771-012-1197-9)

[29] Tian M, Liu R, Gao Z, Chen P, Han H, Wang L, Zhang C, Sun W, Hu Y 2018 Activation mechanism of Fe (III) ions in cassiterite flotation with benzohydroxamic acid collector. Miner. Eng. 119, 31–37. (10.1016/j.mineng.2018.01.011)

[30] Peng H, Luo W, Wu D, Bie X, Shao H, Jiao W, Liu Y 2017 Study on the effect of Fe**^3+^** on zircon flotation separation from cassiterite using sodium oleate as collector. Minerals 7, 108 (10.3390/min7070108)

[31] Xia W, Zhou C, Peng Y 2017 Enhancing flotation cleaning of intruded coal dry-ground with heavy oil. J. Clean. Prod. 161, 591–597. (10.1016/j.jclepro.2017.05.193)

[32] Li F, Zhong H, Zhao G, Wang S, Liu G 2015 Flotation performances and adsorption mechanism of α-hydroxyoctyl phosphinic acid to cassiterite. Appl. Surf. Sci. 353, 856–864. (10.1016/j.apsusc.2015.06.147)

[33] Feng Q, Zhao W, Wen S, Cao Q 2017 Activation mechanism of lead ions in cassiterite flotation with salicylhydroxamic acid as collector. Sep. Purif. Technol. 178, 193–199. (10.1016/j.seppur.2017.01.053)

[34] Suzuki Y 2000 Correlation of C1s chemical state intensities with the O1s intensity in the XPS analysis of anodically oxidized glass-like carbon samples. J. Mater. Sci. 35, 139–146. (10.1023/A:1004835111782)

[35] Nowak P, Laajalehto K, Kartio I 2000 A flotation related X-ray photoelectron spectroscopy study of the oxidation of galena surface. Colloids Surf. A Physicochem. Eng. Asp. 161, 447–460. (10.1016/S0927-7757(99)00214-9)

[36] Ren L, Qiu H, Zhang M, Feng K, Liu P, Guo J, Feng J 2017 Behavior of lead ions in cassiterite flotation using OHA. Ind. Eng. Chem. Res. 56, 8723–8728. (10.1021/acs.iecr.7b02126)

